# Phytoplankton size diversity and ecosystem function relationships across oceanic regions

**DOI:** 10.1098/rspb.2018.0621

**Published:** 2018-05-23

**Authors:** Esteban Acevedo-Trejos, Emilio Marañón, Agostino Merico

**Affiliations:** 1 Systems Ecology Group, Leibniz Centre for Tropical Marine Research (ZMT), Bremen, Germany; 2 Departamento de Biología y Ecología Animal, Universidad de Vigo, Vigo, Spain; 3 Faculty of Physics & Earth Sciences, Jacobs University Bremen, Bremen, Germany

**Keywords:** functional biogeography, trait-based ecology, macroecology, biodiversity ecosystem function relationship, latitudinal gradient, environmentally mediated trade-off

## Abstract

Trait diversity, a key component of biodiversity, mediates many essential ecosystem functions and services. However, the mechanisms behind such relationships at large spatial scales are not fully understood. Here we adopt the functional biogeography approach to investigate how the size composition of phytoplankton communities relates to primary production and export production along a broad latitudinal gradient. Using *in situ* phytoplankton size distribution data and a trait-based model, we find an increase in the average phytoplankton size, size diversity, primary production and export when moving from low to high latitudes. Our analysis indicates that the interplay between spatio-temporal heterogeneities in environmental conditions and a trade-off between the high affinity for nutrients of smaller cells and the ability to avoid predation by larger cells are the main mechanisms driving the observed patterns. Our results also suggest that variations in size diversity alone do not directly lead to changes in primary production and export. The trade-off thus introduces a feedback that influences the relationship between size diversity and ecosystem functions. These findings support the importance of environmentally mediated trade-offs as crucial mechanisms shaping biodiversity and ecosystem function relationships at large spatial scales.

## 1. Introduction

Biodiversity, intended in its broadest sense (i.e. the variety of species, genes and functional traits in an ecosystem), is linked to key ecosystem functions (e.g. primary production, nutrient cycling and decomposition) and thus to ecosystem services that are essential to humans (e.g. food production and climate regulation) [1]. The notable grassland experiments carried out at Cedar Creek [2,3] showed that the increase in grass biodiversity, in terms of number of species and functional groups, leads to greater primary production, better resource utilization and higher ecosystem stability [3]. However, this positive effect of biodiversity on primary production is not fully accepted and some studies [4–6] suggested positive, unimodal or (to a lesser extent) negative relationships between biodiversity and primary productivity.

The debate on the shape of the biodiversity–productivity (BP) relationships continues, while calls to concentrate also on the mechanism driving such relationships have been raised [7]. Recent theoretical work [8] identified two mechanisms shaping BP relationships: (i) a growth–affinity trade-off that permits the coexistence of different species when the environment fluctuates and is spatially heterogeneous, and (ii) a competition–vulnerability trade-off that occurs under the presence of grazers, leading to a predator-mediated complementarity effect between coexisting species. Further theoretical studies by Hodapp *et al*. [9] and Smith *et al*. [10] have highlighted the importance of trade-offs and environmental variability as key mechanisms mediating BP relationships. These studies observed that increases in the mean trait value and in the frequency of environmental disturbances (or in the spatial heterogeneity of resources) lead to an increase in phytoplankton production by means of both complementarity and selection. This suggested a stronger BP relationship when the community mean trait value and environmental variability increased [9,10].

These works [8–10] constitute a theoretical foundation for the use of functional traits and trade-offs in understanding the effects of biodiversity on ecosystem functions. However, the theoretical predictions of these works have not been verified yet by observational evidence of trait distributions at macroecological scales.

The need to better describe, explain and predict large-scale distributions of traits and functions has fostered a new field of research called functional biogeography [11]. This young area of research combines knowledge of traditional fields such as ecology, biogeography and earth systems science, with the aim of studying the distribution of forms and functions of organisms, populations, communities and ecosystems along large spatial scales [11,12]. This new approach uses functional traits as a currency to link different organizational levels (e.g. from organisms to ecosystems [13,14]). For marine phytoplankton, cell size can be an appropriate trait for exploring the large-scale functional biogeography patterns of trait diversity, because of its known relationships with other traits and its strong influence on key ecosystem functions [15–18].

Following the functional biogeography perspective, here we explore the patterns of phytoplankton size diversity and its relationships with community structure and key ecosystem functions along different biogeographic regions of the Atlantic Ocean. We use the trait-based model of phytoplankton community properties (i.e. total biomass, mean size and size diversity) PhytoSFDM [19] in combination with high-resolution size distribution data collected along the Atlantic Meridional Transect (AMT) [20,21]. Our aim is to provide insights into the mechanisms shaping the patterns of phytoplankton size diversity and ecosystem function (specifically, primary production and export) relationships along large spatial scales.

## 2. Methods

### (a) Observations

We used phytoplankton size spectrum data from two cruises that were part of the AMT programme. The cruises took place between September and October 1996 (AMT3) and April and May 1997 (AMT4). In total, we used 43 samples from tropical, subtropical and temperate open ocean areas. The size spectrum of each sample spans 18 size classes (from 0.6 to 80 µm in equivalent spherical diameter, ESD) and for each size class total cell abundance has been determined. Cell abundances were transformed to carbon-based biomass following [22]. Details regarding the processing and handling of samples can be found in [20,21].

For each sample, we quantified the weighted mean size (*S*_w_) and the weighted size variance (*V*_w_) as:2.1
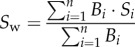
2.2
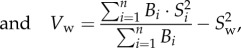
where *B* and *S* are the biomass and the size of each observed size class *i*, for a total number of *n* size classes (*n* = 18 in our case).

### (b) Trait-based model

We used PhytoSFDM [19], which is a trait-based model that describes the phytoplankton community in terms of three macroecological properties: total biomass, mean cell size and size variance (or size diversity). The physical structure of the ocean is simplified into two vertically stacked layers. The upper part represents the well-lit mixed layer of the ocean, where all ecological processes are simulated explicitly, and the bottom part represents the dark, deep oceanic layer, where nutrients are received from, and resupplied to, the upper layer. The essential aspects of the model are the representation of the changing environment and a trade-off that allows different competitive abilities of phytoplankton cell sizes to emerge. The changing environment is represented by four forcing variables: (i) the mixed-layer depth (MLD), (ii) the photosynthetically active radiation (PAR), (iii) the sea surface temperature (SST) and (iv) the concentration of nutrients immediately below the upper mixed layer (*N*_0_). The size-based trade-off emerges from three allometric relationships between phytoplankton cell size and (i) phytoplankton nutrient uptake, (ii) zooplankton grazing and (iii) phytoplankton sinking. The first relationship is based on empirical studies [23] relating phytoplankton cell size with the half-saturation constant for nutrient uptake. The resulting function provides smaller cells with a competitive advantage with respect to larger ones, especially under low nutrient supply (electronic supplementary material, figure S1*a*). In contrast, the second allometric relationship makes smaller phytoplankton cells more susceptible of being grazed by zooplankton than larger cells. This grazing formulation is inspired by meta-analysis of laboratory data [24,25] and is encoded as a Holling Type-II or Monod functional response [26]. In line with these works, zooplankton is represented in our model as a generalist grazer with a prey preference towards smaller sizes (electronic supplementary material, figure S1*b*). The last relationship is related to phytoplankton sinking and is based on Stokes's law [27], which makes large cells sink faster than small cells, even under weak vertical mixing (electronic supplementary material, figure S1*c*). The size-based trade-off in our model emerges from the combination of these three size-scaling processes and the prevailing environmental conditions (nutrient concentrations, grazing pressure and vertical mixing). This creates a competitive environment, or interaction milieu, that drives the mean trait of the phytoplankton community towards values that maximizes phytoplankton fitness under the current environmental conditions. This constitutes a so-called environmentally mediated trade-off [28]. Following previous studies [29–31], the temporal dynamics of the three phytoplankton community properties are computed as follows:2.3
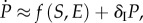
2.4
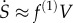
2.5
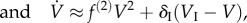
where *P* is the total phytoplankton biomass, *S* is the mean size, *V* is the size diversity, *δ*_I_ is the immigration rate and *V*_I_ is the size diversity of the immigrating community. The net growth, *f*(*S*, *E*), which we assume reflects the fitness of the phytoplankton community, represents the balance between all the gains and losses of phytoplankton biomass, and is a function of the trait (cell size, *S*) and the environmental factors (here generically indicated as *E*, but which includes temperature, light, nutrient availability and grazing pressure). The gains of phytoplankton biomass are captured by three main processes: (i) size-dependent nutrient uptake (as explained above), (ii) light harvesting as a function of depth and light intensity in the upper, well-mixed layer of the ocean [32,33], and (iii) temperature-dependent growth [34]. The losses of phytoplankton biomass depend on the following four processes: (i) size-dependent grazing (as explained above), (ii) size-dependent sinking (as explained above), (iii) mixing losses [35], and (iv) losses other than grazing, sinking and mixing. The superscripts 1 and 2 indicate, respectively, the first and second derivative of net growth *f* with respect to mean size *S*. Immigration, defined by the parameters *δ*_I_ and *V*_I_, is the mechanism that helps to sustain size diversity in the model. Without such a mechanism only an optimal size class would survive due to competitive exclusion [19,36]. The model also includes differential equations for nutrients (*N*), zooplankton (*Z*) and detritus (*D*). A detailed description of the equations, functions and parameters constituting the trait-based model is provided in electronic supplementary material, text 1. The model consists of 17 parameters, four of which (those of the allometric relationships for nutrient uptake and sinking) are fixed to values reported in the literature [23,27]. The remaining 13 are considered as free parameters. The values of the free parameters were adjusted manually, one at a time, to match observations of phytoplankton total biomass, mean size and size diversity along the AMT. We varied these parameters within reasonable ranges reported in the literature by similar modelling studies (electronic supplementary material, table S1).

### (c) Model forcing

PhytoSFDM uses monthly climatological forcing data for MLD, PAR, SST and *N*_0_, obtained from the World Ocean Atlas (MLD, SST and *N*_0_) and the NASA's Ocean Biology Processing Group, MODIS data (PAR) [19]. The monthly forcing was spatially averaged over each 10° × 10° box and then interpolated to obtain daily values [19]. Therefore, each 10° × 10° box is assumed to represent a homogeneous region along the Atlantic Meridional Transect. The effects of lateral advection (although crudely captured in our model by the immigration mechanism) and other spatially explicit physical processes (e.g. fine and large scale turbulence) have been the object of previous works [37,38] and were thus beyond the scope of our study.

### (d) Ecosystem functions

We evaluated two ecosystem functions along the latitudinal diversity gradient, namely gross primary production (GPP) and export. GPP is assumed equal to phytoplankton gross growth (similar to [35]) and export is the sum of all outward fluxes in the model (similar to [35]; see also electronic supplementary material, text 1). Observations of GPP were based on simulated *in situ* incubation experiments that measured ^14^C fixation rate as in [20,21]. Hourly carbon fixation rates were multiplied by photoperiod length to obtain GPP estimates, assuming that dark respiration is 20% of daylight primary production and that dissolved organic carbon production accounts for 20% of total primary production [39]. Where necessary, the data were log-transformed to meet normality assumptions, and all statistical analyses were performed using R (v. 3.3).

### (e) Simulations

**Figure 1.**
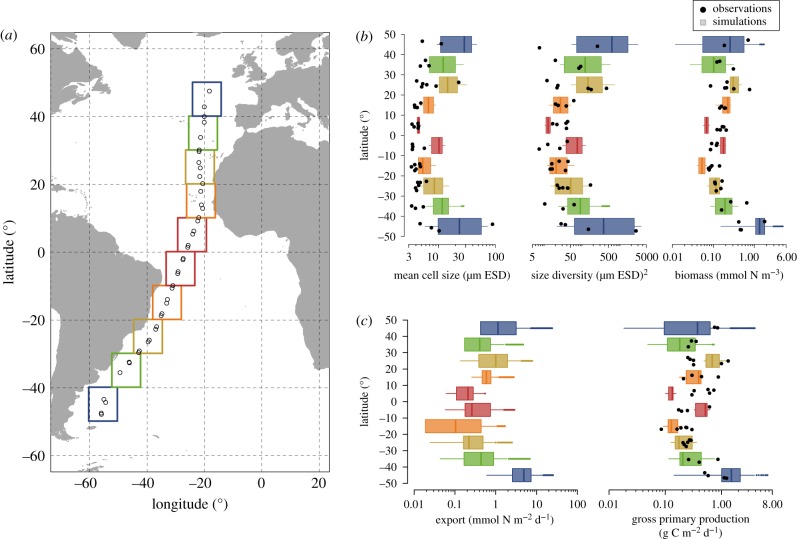
Spatio-temporal patterns of phytoplankton community properties and ecosystem functions. (*a*) Geographical locations (empty circles) of the phytoplankton size distribution data observed along the AMT with coloured squares indicating the 10° × 10° regions considered for the trait-based model application. (*b*) Phytoplankton community properties along the AMT. Box plots represent the temporal variability of a typical annual cycle predicted by the trait-based model at each 10° × 10° location, with vertical lines representing the median, hinges of the box representing the first and third quartile, whiskers representing the 95% confidence interval of the distribution of the specific variable captured by the model (mean cell size, size diversity, biomass, gross primary production and export), and small coloured dots representing outliers. The black dots represent *in situ* observations. (*c*) Ecosystem functions represented by primary production and export along the latitudinal transect.

The model is run at each 10° × 10° location of the AMT (figure 1*a*) with unchanged parametrization. Therefore, the functional biogeography patterns emerge solely from the environmental forcing, which is specific to each 10° × 10° location, and the size-based trade-off. The model is then initialized at each location with the same values for the state variables *P*, *Z*, *D*, *S* and *V*. *N* is initialized with the local annual average of nutrient concentration immediately below the mixed layer. The model was then run over repeated annual cycles for 10 years until a steady-state solution was reached. For our analysis, we used the results of the last year at each location. Rather than plotting the results of the model simulations in a traditional way, as functions of time, we used box plots. This allowed us to illustrate, simultaneously, the temporal variability within a typical annual cycle (height of the box plots) and the spatial variability along the latitudinal transect (difference between box plots).

### (f) Sensitivity analyses

We conducted a series of sensitivity analyses to investigate how the model predictions depend on: (i) changes in immigration rate (*δ*_I_) and diversity of the immigrating community (*V*_I_), (ii) fixed mean cell sizes for the two main size-dependent processes of phytoplankton nutrient uptake and zooplankton grazing, and (iii) fixed nutrient supply values (*N*_0_ in our model).

In the first sensitivity test, we assessed the impacts that *δ*_I_ and *V*_I_ have on size diversity, gross primary production and export. For this we systematically varied the parameters by ±25% and ±50%, and evaluated the changes in model results using a sensitivity index (*γ*):2.6
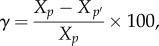
where *X_p_* is the value of the variable *X* obtained with the reference parameter (as reported in electronic supplementary material, table S1) and *X_p′_* is the value of the variable *X* obtained when the parameter has been varied by ±25% or ±50%. In total, we evaluated 25 possible combinations of the parameters *δ*_I_ and *V*_I_. For simplicity, we reduced all possible combinations of the parameters *δ*_I_ and *V*_I_ into nine possible cases (electronic supplementary material, text 5 and table S2).

With the second sensitivity test we demonstrate how the size-based trade-off operates to produce the patterns predicted by our model. We consider three cell sizes—3, 10 and 30 µm ESD—representing, respectively, the minimum size observed in the data, an arbitrary intermediate size and the maximum size observed in the data. We first run the model by fixing the cell size for nutrient uptake (equation (11) in electronic supplementary material, text 1) to each of the three cell sizes (small, intermediate and large), but allowing all the other size-dependent processes to change dynamically as a function of the varying cell size (as computed from equation (2.4)). We then repeat the same procedure for zooplankton grazing (equation (12) in electronic supplementary material, text 1). We finally compare the model results obtained with these tests with the observations of mean cell size, size diversity and biomass.

With the third sensitivity test, we elucidate the role that nutrient supply and its variability play in producing the observed latitudinal patterns of mean cell size, size diversity and total biomass. For this we fixed the nutrient supply (*N*_0_ in our model) to low (0.1 mmol N m^−3^), intermediate (2 mmol N m^−3^) and high (10 mmol N m^−3^) values, but allowed the other environmental factors (MLD, PAR and SST) to vary during the simulations. We then compared the model predictions with the observations.

## 3. Results

Our trait-based model produces patterns of phytoplankton community properties (total biomass, mean cell size and size diversity) and ecosystem functions (gross primary production and export) that are in agreement with *in situ* data across a broad range of biogeographic regions along the AMT (figure 1*a*; electronic supplementary material, text 2 and figure S2). Specifically, all community properties decrease when moving from high latitudes to the equator (figure 1*b*). Consistently, gross primary production and export decrease equatorward (figure 1*c*).

**Figure 2.**
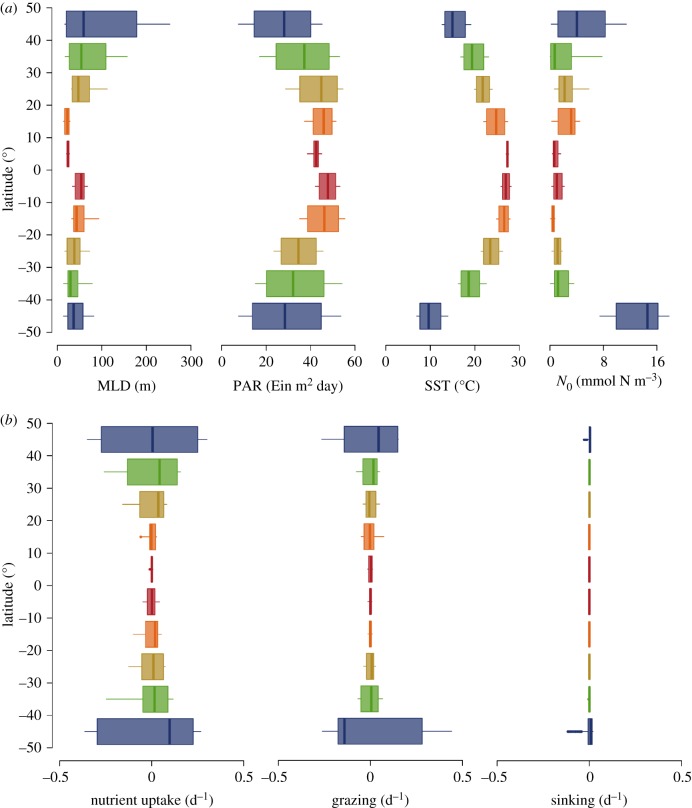
Spatio-temporal patterns of environmental variables and size-scaling processes. (*a*) Annual variability of mixed layer depth (MLD), photosynthetic active radiation (PAR), sea surface temperature (SST) and concentration of nutrients immediately below the upper mixed layer (*N*_0_), used as forcing to the model. (*b*) Variability in the size-dependent processes that produce the trade-off in the model, with box plots representing anomalies from annual averages (i.e. annual mean minus daily values). In the box plots the vertical line represents the median, the hinges of a box are the first and third quartile, the whiskers represent the 95% confidence interval of the distribution, and the small coloured dots are outliers.

The simulated functional biogeography patterns emerge from the interplay of two model features: (i) the spatial and temporal heterogeneities of the environmental conditions (figure 2*a*), and (ii) the size-dependent processes of nutrient uptake, grazing and sinking that constitute the environmentally-mediated trade-off (figure 2*b*). The annual variability (indicated by the height of the box plots) of the environmental conditions decreases when moving from high to low latitudes, whereas the average magnitude of the environmental variables increases (PAR and SST) or decreases (MLD and *N*_0_) when moving equatorward (figure 2*a*). Among the size-dependent processes, nutrient uptake and grazing vary more and show higher mean values in temperate areas than in the tropics (figure 2*b*). Sinking shows negligible spatio-temporal variations in all locations compared to nutrient uptake and grazing (figure 2*b*). However, sinking does vary through latitude because it is related to the variations in mean cell size (electronic supplementary material, text 3 and figure S3). This low variability in sinking rates depends mainly on model assumptions regarding (i) the simplified physical setting and (ii) the selected allometric relationship between sinking speed and cell size [27].

**Figure 3.**
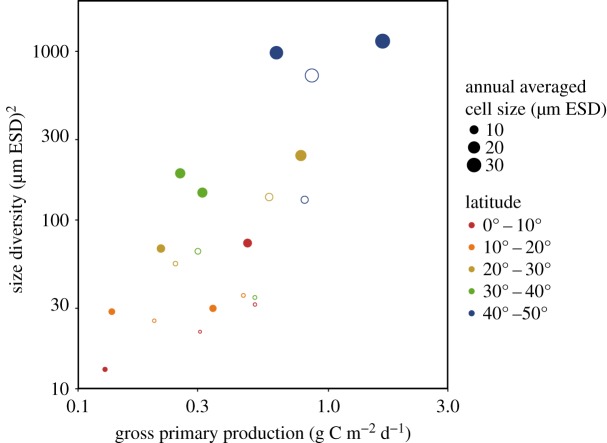
Relationship between phytoplankton size diversity and primary production along the AMT. The empty and filled circles represent, respectively, the annual averaged *in situ* observations and model results. The colour of the dots represents latitude (in absolute terms) for each 10° × 10° region, as specified in figure 1. The size of the points indicates the annual averaged cell size of the phytoplankton community.

The annually averaged community properties and ecosystem functions show decreasing trends from the temperate areas to the tropics (figure 3). In addition, the annually averaged size diversity and gross primary production are positively correlated (model results: Pearson *r*: 0.835, d.f.: 8, 95% CI: 0.434–0.960; *in situ* observations: Pearson *r*: 0.778, d.f.: 8, 95% CI: 0.291–0.944; figure 3). Regions with smaller mean cell sizes, low size diversity and low primary production, corresponding to tropical and subtropical locations, cluster together in the lower-left corner of figure 3, whereas regions with larger mean cell sizes, high size diversity and high primary production, corresponding to temperate locations, fall near the upper-right corner. A similar pattern is also observed in the relationship between size diversity and export (Pearson *r*: 0.945, d.f.: 8, 95% CI: 0.778–0.987; electronic supplementary material, text 4 and figure S4).

Immigration (defined by the parameters *δ*_I_ and *V*_I_, respectively, immigration rate and size diversity of the immigrating community) has an influence on the size diversity simulated by the model. A sensitivity analysis of size diversity, GPP and export to systematic variations of these parameters by ±25% and ±50% suggests a strong impact on phytoplankton size diversity and a moderate impact on GPP and export (electronic supplementary material, text 5 and figure S5). More specifically, higher immigration rates (by about 50% more than the reference value) and larger size diversities (by about 50% more than the reference value) of the immigrating community tend to increase the average size diversity, GPP and export by about 80%, 3% and 1%, respectively. In contrast, lower immigration rates (by about 50% less than the reference value) and smaller size diversities (by about 50% less than the reference value) of the immigrating community tend to decrease average size diversity, GPP and export by about 55%, 3% and 1%, respectively. This sensitivity analysis also suggests that phytoplankton size diversity is more sensitive to changes in these parameters in the temperate regions (with either an increase or a decrease from the reference value by up to 100%) than in the tropics (electronic supplementary material, figure S5). GPP and export do not show similar pronounced patterns of sensitivities to *δ*_I_ and *V*_I_ because these ecosystem functions are not solely influenced by immigration but also by the interplay between the size-based trade-off and the environmental conditions (mainly nutrient supply and its variability).

We conducted two additional sensitivity tests to better disentangle the effects of the size-based trade-off and nutrient supply and its variability on the model results. In the first test, we performed model runs at fixed mean cell sizes, first for phytoplankton nutrient uptake (electronic supplementary material, figures S6 and S7) and then for zooplankton grazing (electronic supplementary material, figures S8 and S9). A low, fixed mean cell size in the nutrient uptake function produces high nutrient uptake rates relative to grazing rates (electronic supplementary material, figure S7*a*–*c*), thus leading to model results that overestimate the observations (electronic supplementary material, figure S6*a*–*b*). Similarly, fixing the grazing formulation to large cell sizes produces lower grazing rates relative to nutrient uptake (electronic supplementary material, figure S9*g*–*i*) and thus model results that overestimate the observations (electronic supplementary material, figure S8*g*–*i*). These tests do not produce any appreciable effect on phytoplankton total biomass, although the annual variability (i.e. height of the box) increases when we use a small mean cell size and a large mean cell size for, respectively, nutrient uptake, and grazing (electronic supplementary material, figures S6 and S8). This increased annual variability is also visible for mean cell size and size diversity, although to a lesser extent than for total phytoplankton biomass (compare first two columns with third column in electronic supplementary material, figures S6 and S8). These tests indicate that the trade-off emerging from the size-dependent processes operates in concert with the current environmental conditions to produce the observed latitudinal patterns.

In the second test we analysed the role that nutrient supply and its variability have on the predicted community size structure along the Atlantic Meridional Transect (electronic supplementary material, figure S10). Under low nutrient supply, the model produces smaller mean sizes, lower size diversities and lower biomasses than the observations (electronic supplementary material, figure S10*a*–*c*). In contrast, under high nutrient supply, the model produces relatively higher mean cell sizes, higher size diversities and higher biomasses than the observations and in relation to the previous case (compare panels *a*–*c* with *d*–*f* and *g*–*i* in electronic supplementary material, figure S10). This effect is amplified in temperate locations, where the seasonal variability of the mixed layer depth is stronger (blue boxes in electronic supplementary material, figure S10). In temperate locations the variabilities of mean cell size, size diversity and biomass are also more pronounced. In contrast, an increase in nutrient supply in tropical locations, where the seasonal variability is low (red boxes in electronic supplementary material, figure S10), produces model results characterized by higher mean cell size, size diversity and biomass, but with less pronounced variability. Therefore, the increase in the nutrient supply or in its variability has a positive effect on phytoplankton community size structure, allowing for communities with larger and more variable cell sizes to prevail.

## 4. Discussion

We investigated the mechanisms producing functional biogeography patterns of marine phytoplankton communities in the Atlantic. Using *in situ* size-distribution data and a trait-based model we showed an equatorward decreasing trend in phytoplankton biomass, mean size, size diversity, gross primary production and export (figure 1). These latitudinal patterns are congruent with the current general understanding of phytoplankton biogeography based on *in situ* [20,21] and remotely sensed [40,41] data, observations of functional groups based on pigment concentrations [42], and different trait-based modelling applications [43,44].

We found that the main mechanisms driving the observed patterns are spatio-temporal heterogeneities of the environmental conditions and variations in the relative influence of bottom-up (nutrient concentration) versus top-down (zooplankton abundance) controls driven by the size-based trade-off. At temperate latitudes, for example, the environmental conditions change seasonally, thus varying nutrient uptake and grazing pressure, which in turn drive changes in (i) phytoplankton biomass, (ii) relative distributions of larger cells versus smaller cells and (iii) size diversity. Conversely, in tropical and subtropical areas, environmental conditions remain relatively stable during the year and the interplay between nutrient uptake and grazing pressure produce narrow size distributions, characterized by small mean cell size and low biomass. Our analysis thus strengthens the view that the broad range of environmental variability in the temperate regions sustains phytoplankton communities with higher biomass, larger mean cell sizes, higher size diversity, and higher GPP and export (figure 1*b*,*c*) than in the tropical regions. Here we showed that these patterns are generated by a trade-off, which balances the higher affinity for nutrients of smaller cells against the ability to avoid predation by larger cells (see Methods section; electronic supplementary material, text 1 and figure S1). This mechanism of combined size-dependent, bottom-up (through a nutrient utilization trait) and top-down (through size-selective grazing) controls has been previously suggested [45–47] in models that described the phytoplankton community with a discrete number of size classes [48–50]. Our results suggest that observations along the AMT can be explained by the combined effect of size-scaled nutrient uptake and size-selective grazing, producing positive correlations between community biomass, larger mean cell size and higher size diversity (figure 1).

We showed that the observed patterns of community size structure along the AMT are strongly influenced by nutrient supply and its variability (here intended as the variability within a typical year cycle at each location; figure 2*a*). It is well known that a surge in the nutrient supply tends to increase the number of larger cells in a phytoplankton community without necessarily decreasing the number of small cells, which results in higher diversity in terms of cell sizes [45–50]. This is supported by observations in coastal regions [51] and open oceans [52]. Additionally, support comes from theoretical studies suggesting that constant nutrient supply selects for smaller organisms [53,54], while variable nutrient supply promotes a community with larger and more diverse organisms, especially at intermediate frequencies (around 30 to 40 days) of nutrient pulses [54] or under fine-scale turbulence (in eddies of around 300 to 10 000 µm in length) [38]. Our work, which integrates, for the first time and over broad biogeographic scales, model and *in situ* size spectrum observations, is consistent with these views showing that an increase in nutrient supply (*N*_0_ in our model) and its variability (i.e. in terms of relative differences between temperate and tropical regions, blue boxes in figure 2*a*) promotes communities with higher mean cell size and higher size diversity (electronic supplementary material, figure S10).

Size-selective grazing is formulated in our model as a generic zooplankton grazer with a preference towards smaller cells (electronic supplementary material, figure S1*b*). In recent years, however, significant efforts have been made to increase the level of detail of the zooplankton component in ecosystem models. Approaches are numerous and include the consideration of different zooplankton functional types, different size classes, and different feeding preferences and strategies [44,49,55–57]. Accounting for such details in the grazing formulation can have a considerable impact on model results. For example, different zooplankton functional types, distinguished by variable feeding preferences, can promote the diversity of phytoplankton communities without requiring mechanisms such as immigration for sustaining phytoplankton diversity [44,49,58]. Implementing more elaborated grazing mechanisms and processes is a natural step forward in the development of ecosystem models. However, a consistent representation of different grazing strategies remains an aspect under development [59–61]. Thus, our grazing formulation represents the simplest approach possible capable of reproducing large scale ecological patterns of phytoplankton community structure along the AMT.

Theoretical studies suggest that environmental disturbances mediate not only the size structure of phytoplankton communities but also their ecosystem functions. For example, a work based on a spatially explicit resource competition model [9] showed a complementarity effect on ecosystem functions when functionally different species coexist in a landscape with heterogeneous resource supply. They suggested that selection effects are maximized when broad trait variations coincide with narrow ranges of resource supply ratios, which favour a limited number of functionally similar species. Another work, based on an explicit size-based model [10], found that primary productivity increases at high disturbances and intermediate size diversity, suggesting that disturbance levels can influence the relationship between size diversity and ecosystem functions. Our results, based on an approach that combines *in situ* data with trait-based modelling simulations, thus provide further evidence in support of these theoretical predictions by showing a high degree of complementarity (based on a larger variety of coexisting cell sizes) in highly productive temperate regions of the Atlantic, which are characterized by variable environmental conditions. In contrast, we observe stronger selection for narrower ranges of cell sizes in less productive tropical regions of the Atlantic, areas characterized by stable environmental conditions.

The positive relationship we found between the annually averaged size diversity and primary production is also in agreement with previous theoretical studies [8,62], although they considered a different component of biodiversity (i.e. species richness), whereas our work is focused on size diversity. Nonetheless, both the results of these previous works and our results are explained by the same mechanism (i.e. a trade-off between nutrient utilization and vulnerability to grazing). This commonality, which is not easily deducible, especially considering the different assumptions and approaches used in the different models, points at the robustness and generality of this trade-off in producing similar patterns for different components of biodiversity. Ultimately, our work provides evidence that an environmentally mediated trade-offs [28] are a key feature that shapes phytoplankton community size composition, size diversity and ecosystem functions at broad biogeographic scales.

## Supplementary Material

Supplementary Material

## Code and data accessibility

The trait-based model PhytoSFDM is coded in Python. Python classes are used to describe the phytoplankton community properties and other state variables and to retrieve and implement the environmental forcing. PhytoSFDM is provided as open source software under the GNU General Public License, version 2, and can be downloaded from GitHub (https://github.com/SEGGroup/PhytoSFDM), Zenodo (https://doi.org/10.5281/zenodo.49849) or PyPi (https://pypi.python.org/pypi/PhytoSFDM). The phytoplankton size distribution data along the Atlantic Meridional Transect are available at the Dryad Digital Repository (http://dx.doi.org/10.5061/dryad.41pm8qf).
