# Supplementary material for: Phytoplankton size diversity and ecosystem function relationships across oceanic regions
Source: Proc Biol Sci. 2018 May 23;285(1879):20180621. doi: 10.1098/rspb.2018.0621 (PMC5998115; doi:10.1098/rspb.2018.0621)
Supplement: Supplementary Material [file rspb20180621supp1.pdf]

Supplementary Material

Phytoplankton size diversity and ecosystem function relationships across different oceanic regions. Esteban Acevedo-Trejos* [a], Emilio Marañón[b], and Agostino Merico[a, c]

[a] Systems Ecology Group, Leibniz Centre for Tropical Marine Research (ZMT), Bremen, Germany

[b] Departamento de Biología y Ecología Animal, Universidad de Vigo, Vigo, Spain

[c] Faculty of Physics & Earth Sciences, Jacobs University Bremen, Bremen, Germany

* Corresponding author: esteban.acevedo@leibniz-zmt.de

Supplementary Text 1. Trait-based model description

To investigate the mechanisms driving the latitudinal patterns of the size diversity and ecosystem function relationship we used the Phytoplankton Size and Functional Diversity Model (PhytoSFDM) [1]. The model is a set of differential equations describing the temporal evolution of phytoplankton total biomass (P), mean cell size (S), size variance (or size diversity, V), inorganic nutrient (N), zooplankton (Z), and detritus (D). The ocean structure is simplified into two vertically stacked layers. The biological and ecological processes are simulated in the upper layer, which is assumed to be homogeneously mixed with a seasonally varying depth (M) implemented as external forcing. The deeper layer receives/supplies nutrients from/to the upper layer. The exchange between the two layers (K) is a function of the mixed layer depth as follows [2,3]:

$$K = \frac{\kappa + h^{+}(t)}{M(t)}, \qquad (1)$$

where κ represent the diffusive mixing across the thermocline and $h^{+}(t)$ is a function that describes the entrainment and detrainment of material [2,3]. The latter is given by $h^{+}(t)=max(h(t),0)$, with $h(t)= dM(t)/dt$.

The coupled temporal dynamics of the six state variables in PhytoSFDM is simulated as follows:

$$\dot{N} = \underbrace{-P \cdot \mu_P \cdot F(T) \cdot H(I) \cdot U(S,N)}_{P\ gross\ growth} + \underbrace{\delta_D \cdot D}_{remineralization} + \underbrace{K \cdot [N_0 - N]}_{mixing} + \underbrace{\epsilon_N}_{higher\ order\ correction}, \quad (2)$$

$$\dot{P} = \underbrace{f(S,E)}_{fitness\ function} + \underbrace{P \cdot \delta_I}_{immigration} + \underbrace{\epsilon}_{higher\ order\ correction}, \quad (3)$$

$$\dot{Z} = \underbrace{\delta_Z \cdot \mu_Z \cdot G(S,P) \cdot P \cdot Z}_{assimilated\ Z\ grazing} - \underbrace{m_Z \cdot Z^2}_{Z\ mortality} - \underbrace{\frac{h(t)}{M(t)} \cdot Z}_{mixing} + \underbrace{\epsilon_Z}_{higher\ order\ correction}, \quad (4)$$

$$\dot{D} = \underbrace{[1 - \delta_Z] \cdot \mu_Z \cdot G(S,P) \cdot P \cdot Z}_{unassimilated\ Z\ grazing} + \underbrace{m_P \cdot P}_{other\ P\ losses} + \underbrace{m_Z \cdot Z^2}_{Z\ mortality} - \underbrace{\delta_D \cdot D}_{remineralization} - \underbrace{K \cdot D}_{mixing} + \underbrace{\epsilon_{D,}}_{higher\ order\ correction} \quad (5)$$

$$\dot{S} = V \cdot \underbrace{\frac{\partial f(S,E)}{\partial S}}_{first\ derivative\ of\ the\ fitness\ function\ with\ respect\ to\ the\ trait}, \quad (6)$$

$$\dot{V} = V^2 \cdot \underbrace{\frac{\partial^2 f(S,E)}{\partial^2 S}}_{second\ derivative\ of\ the\ fitness\ function\ with\ respect\ to\ the\ trait} + \underbrace{\delta_I \cdot [V_I - V_S]}_{Immigrating\ source\ of\ size\ variance}, \quad (7)$$

Fitness function. The phytoplankton fitness function *f(S,E)* represents the net growth of phytoplankton and is a function of mean cell size S and the relevant environmental variables E, as follows:

$$f(S,E) = P \cdot [\mu_P \cdot F(T) \cdot H(I) \cdot U(S,N) - \mu_Z \cdot G(S,P) \cdot Z - v(S,M) - m_P - K], \quad (8)$$

where the first term accounts for the gross growth of phytoplankton and is described by the product of maximum growth rate μ_P, the temperature-dependent growth term according to Eppley's formulation [4] $F(T) = e^{0.063 \cdot T}$, the light limiting term according Steel's formulation and Beer-Lambert law for the decay of light I with depth z [5,6]:

$$H(I) = \frac{1}{M(t)} \cdot \int_0^M \left[\frac{I(z)}{I_s} \cdot e^{\left(1 - \frac{I(z)}{I_s}\right)} \right] dz, \quad (9)$$

$$I(z) = I_0 \cdot e^{-k_w \cdot z}, \quad (10)$$

where I_s is the light level at which photosynthesis saturates, $I(z)$ is the irradiance at depth z, I_0 is the light at surface, i.e. PAR (considered as external forcing to the model), and k_w is the generic light extinction coefficient.

Nutrient limitation is simulated with a Monod function [7] with an size-scaled half-saturation constant (K_N) based on observed allometric relationships [8]:

$$U(S, N) = \frac{N}{N + K_N} = \frac{N}{N + \beta_U \cdot S^{\alpha_U}}, \quad (11)$$

where β_U and α_U are respectively the intercept and slope of the K_N allometric function. This size-dependent process tends to favour smaller over larger organisms, especially, under nutrient limited conditions (Supplementary Figure 1).

The loss term $G(S,P)$ is the size-selective grazing, inspired by meta-analyses of laboratory data [9,10] and encoded as a Holling Type-II or Monod functional response [11]. This formulation considers the zooplankton as a generic group with a specific feeding preference that depends on the slope α_G of a power law function with an intercept of 1 [1,12], and half saturation constant of K_P:

$$G(S, P) = \frac{S^{\alpha_G}}{P + K_P}. \quad (12)$$

In line with previous work [8] our grazing formulation represents a generic grazer with a preference towards smaller phytoplankton cells (Supplementary Figure 1B).

The size-dependent process *v(S,M)* describes sinking as a function of size S and depth of the mixed layer M, using the allometric relationship reported by [13], which depends on the intercept β_v and slope of the α_v:

$$v(S,M) = \frac{\beta_v \cdot S^{\alpha_v}}{M(t)}. \tag{13}$$

This sinking formulation favors smaller over larger organisms, making larger cells to sink faster even under weak vertical mixing (See figure 1C).

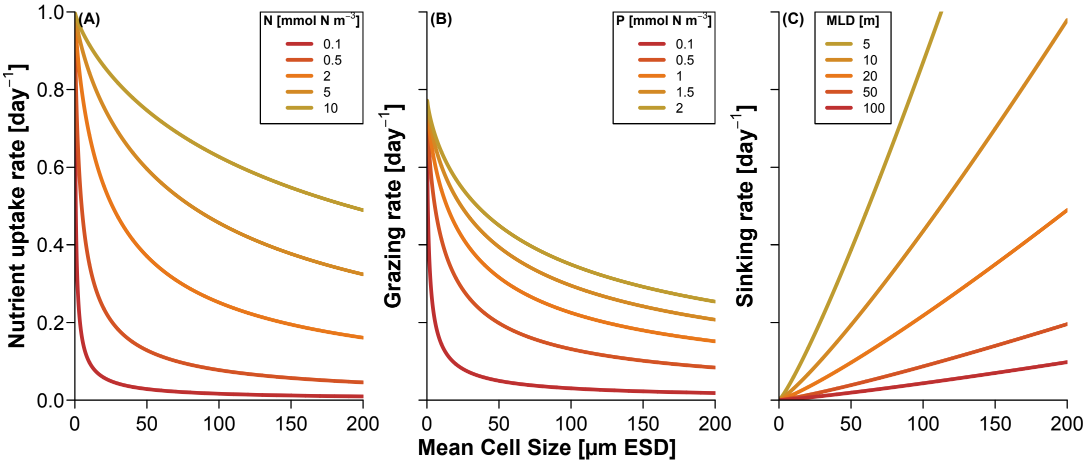

Supplementary Figure 1. The size-based trade-off. The trade-off emerges from three allometric relationships between phytoplankton cell size and A) nutrient uptake, B) zooplankton grazing, and C) phytoplankton sinking.

Last, the term K quantifies the losses due to mixing and the term m_P accounts for phytoplankton losses other than grazing and mixing.

Derivatives of the fitness function and higher order correction terms. The phytoplankton community described here follows the adaptive dynamics approach in which changes of a characteristic trait (phytoplankton cell size in our case) are approximated with a Taylor series expansion and a moment closure technique [14–16]. The respective first and second order derivate of the fitness function f with respect to the trait S are solved in PhytoSFDM using the python library for symbolic algebra sympy. The higher corrections terms ε, ε_N, ε_Z ε_D accounts for higher order moments resulting from the moment closure technique and have little impact on the model dynamics [14–16].

Other processes. The remaining processes close the recirculation of biomass through the system by accounting for remineralization of material from the detritus to nutrients (i.e.: $\delta_D \cdot D$), the predation of higher trophic levels on the modelled zooplankton (i.e.: $m_Z \cdot Z^2$), and losses of N (i.e.: $K \cdot [N_0 - N]$), D (i.e.: $K \cdot D$) and Z (i.e.: $h(t)/M(t) \cdot Z$) due to mixing.

Supplementary Table 1. Parameter definitions, their units, and their default values. The sources marked with the symbol § were tune-up manually and allowed to vary within the range reported in the literate for similar ecosystem models, to improve the model to data fit.

Definition	Symbol (units)	Value	Source
Diffusive mixing across the thermocline	κ (m day^{-1})	0.1	[2,17,18] §
Light attenuation constant	k_w (m^{-1})	0.1	[2,17,19] §
Optimum irradiance	I_s (E m^{-2}day^{-1})	30	§
P max growth rate	μ_P (day^{-1})	1.5	[2,17–21] §
P mortality rate	m_P (day^{-1})	0.05	[2,17–21] §
Z grazing rate	μ_Z (day^{-1})	1.1	[2,17–22] §
Z mortality rate	m_Z (day^{-1})	0.3	[2,17–22] §
P half-saturation	K_P (mmol N m^{-3})	0.1	[2]§
P assimilation coefficient	δ_Z (-)	0.31	[2,17,19] §
Mineralization rate	δ_D (day^{-1})	0.1	[2,17–19] §
Immigration rate	δ_I (mmol N day^{-1})	0.008	§
Slope for allometric grazer preference	α_G ([µmESD]$^{-1}$)	-0.75	[23,24] §
Slope of the half-saturation allometric function	α_U (mmol N m^{-3} $[\mu mESD]^{-1}$)	0.81	[8]
Intercept of the half-saturation allometric function	β_U (mmol N m^{-3})	0.14275	[8]
Slope of the sinking speed allometric function	α_v (m day^{-1} [µmESD]$^{-1}$)	1.17	[13]
Intercept of the sinking speed allometric function	β_v (mday^{-1})	0.01989	[13]
Initial N	N_0 (mmol N m^{-3})	*	§
Initial P	P_0 (mmol N m^{-3})	0.1	§
Initial Z	Z_0 (mmol N m^{-3})	0.1	§
Initial D	D_0 (mmol N m^{-3})	0.01	§
Initial S	S_0 (Ln µmESD)	2.94	§
Initial V / Size variance of immigrating P	V_0 (Ln [µmESD]^2)	5.0	§

* The initial conditions for N are calculated as the annual average of the concentration of nutrients immediately below the mixed layer at each specific 10° by 10° location.

Supplementary Text 2. Comparison of *in situ* and modelled size distributions.

We calculated the probability density functions (PDFs) of each observation across the Atlantic Ocean (Supplementary Figure 2), which show a positively skewed distribution with a peak typically centred between 5 and 10 µm ESD (Supplementary Figure 2A). These distributions are mainly unimodal but one

observation at 40°S-50°S hints at bimodality (Supplementary Figure 2K). We compared the PDFs of the observations with the annual estimate of the model at each 10° by 10° location, from the northernmost location (Supplementary Figure 2B) to the southernmost location (Supplementary Figure 2K). Since the data consisted of punctual observations at specific set of environmental conditions and our modelling approach uses climatologically averaged environmental forcing, we used the annual averages of the mean size and size variance at each location to reconstruct the PDF from the model results (Supplementary Figure 2B to 2K black line). Although, these limitations made it difficult to evaluate the size distribution patterns at finer temporal scales, we can see that our model is able to capture, if only qualitatively, the prominent features of the macroecological patterns and the overall size variability observed across the latitudinal transect.

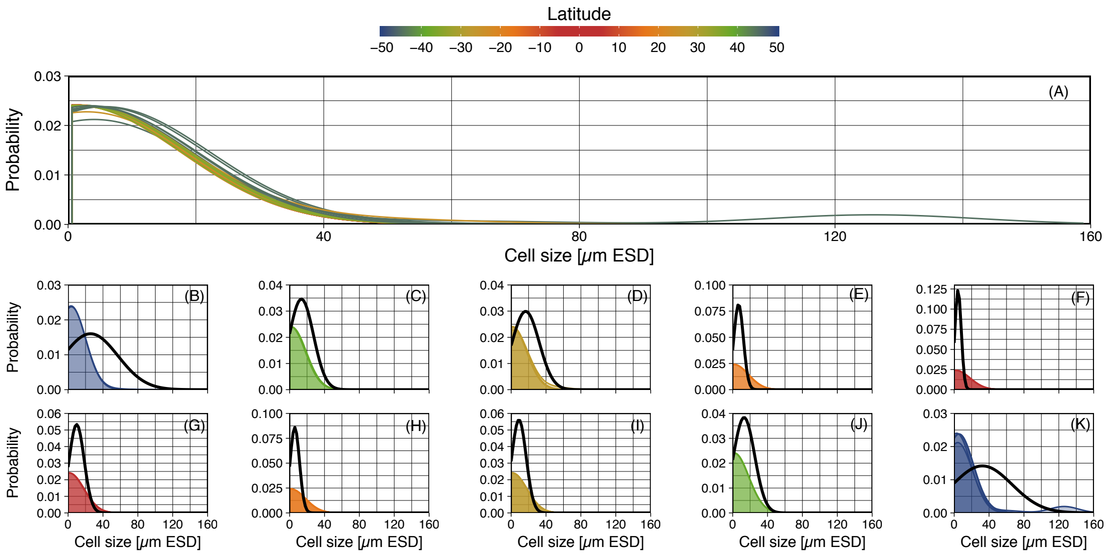

Supplementary Figure 2. Comparison of probability density functions (PDF) between the size spectrum observations and the trait-based model predictions across a latitudinal transect in the Atlantic Ocean. (A) PDFs for each size spectrum observations using a kernel density estimate. (B) to (K) show PDFs of *in situ* observations (colour areas) and model prediction (black line) at each 10° by 10° location (see Figure 1 in main manuscript for reference on the locations), from northernmost (B) to southernmost (K) location. The model PDF is estimated using the annual averaged mean size and size variance at each location and it is based on a truncated normal density function (normal distribution truncated at 0 μm ESD).

Supplementary Text 3. Spatiotemporal patterns of size dependent phytoplankton sinking.

The figure below shows the spatiotemporal pattern of sinking across the latitudinal transect and its relationship with mean cell size at each 10° by 10° location. Sinking shows a similar latitudinal pattern as the two other size dependent processes, i.e. nutrient uptake and grazing, although the losses of this process are negligible compare to the other two. It is also clear that the sinking rate are related to the average cell size of the phytoplankton community as shown in the inset plot in the below figure.

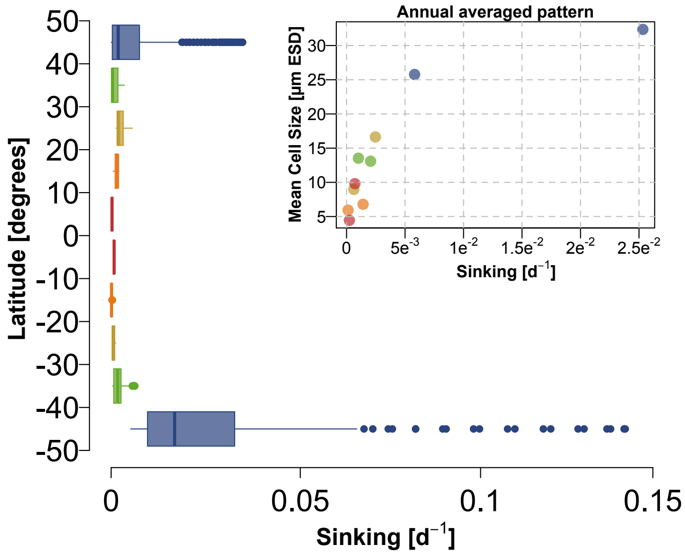

Supplementary Figure 3. Spatiotemporal patterns of size dependent phytoplankton sinking. Boxplots represent the temporal variability of a typical annual cycle predicted by the trait-based model at each 10° by 10° location, with vertical lines representing the median, hinges of the box representing the first, and third quartile, whiskers representing the 95% confidence interval, and small colored dots representing outliers. The inset plot titled "Annual averaged pattern" shows the relationship between mean cell size and sinking rates at each location, where the colour of the dots correspond to the colour of the boxplot.

Supplementary Text 4. Size diversity and export relationship across the latitudinal gradient.

The figure below shows the annual averaged size diversity and the export produced by the model. Export accounts for all outfluxes, i.e. mixing and sinking terms. As described in the article, not only primary production but also export shows a positive correlation with size diversity along the Atlantic Meridional Transect.

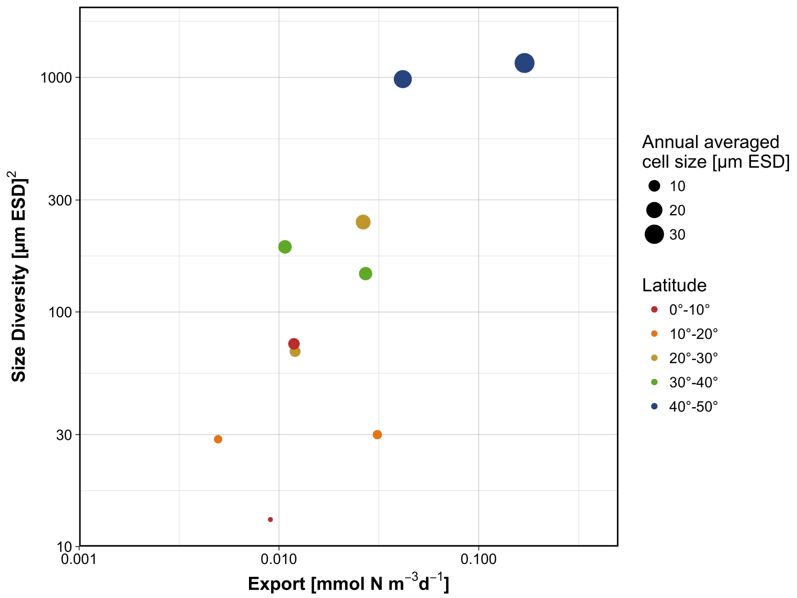

Supplementary Figure 4. Relationship between size diversity and export as calculated from trait based model. The colour of the dots represents latitude (in absolute terms) for each ten degree region as specified on Figure 1. The size of the points illustrates the annual averaged cell size of the phytoplankton community.

Supplementary Text 5. Sensitivity Analyses

Here we summarized the results of three sensitivity tests. The first sensitive test is aimed at assessing the impact that the parameters δ_I and V_I have on size diversity, gross primary production, and export (Supplementary Figure 5). For this a categorization of the 25 possible combinations into nine categories was implemented to simplify the analysis of the results (Supplementary Table 2). The second sensitivity test evaluates how the size-based trade-off operates to produce the predicted latitudinal patterns in our model. We did this by fixing the mean cell size to three values first for nutrient uptake (Supplementary Figure 6 and 7) and then for grazing (Supplementary Figure 8 and 9), while letting all the other size dependent process to vary dynamically. In the third sensitivity test we evaluate the role that nutrient supply and its variability has in producing the observed latitudinal patterns. For this we fixed the nutrient supply (N_0 in our model) to three values: low (0.1 mmol N m^{-3}), intermediate (2 mmol N m^{-3}), and high (10 mmol N m^{-3}), but we allowed the other environmental variables (MLD, SST and PAR) to vary during the simulation according to their respective values at each specific location (Supplementary Figure 10).

Supplementary Table 2. Categorization of results used in the sensitivity analysis. For simplicity we reduced the 25 possible combinations of the parameters δ_I and V_I into nine combinations. Considering only if the parameters increase (superscripted plus sign), decrease (superscripted minus sign) or are equal to the reference value (no superscripted sign).

	$\delta_I^{-50\%}$	$\delta_I^{-25\%}$	δ_I	$\delta_I^{+25\%}$	$\delta_I^{+50\%}$
$V_I^{-50\%}$	$\delta_I^- V_I^-$		$\delta_I V_I^-$	$\delta_I^+ V_I^-$	
$V_I^{-25\%}$					
V_I	$\delta_I^- V_I$		$\delta_I V_I$	$\delta_I^+ V_I$	
$V_I^{+25\%}$	$\delta_I^- V_I^+$		$\delta_I V_I^+$	$\delta_I^+ V_I^+$	
$V_I^{+50\%}$					

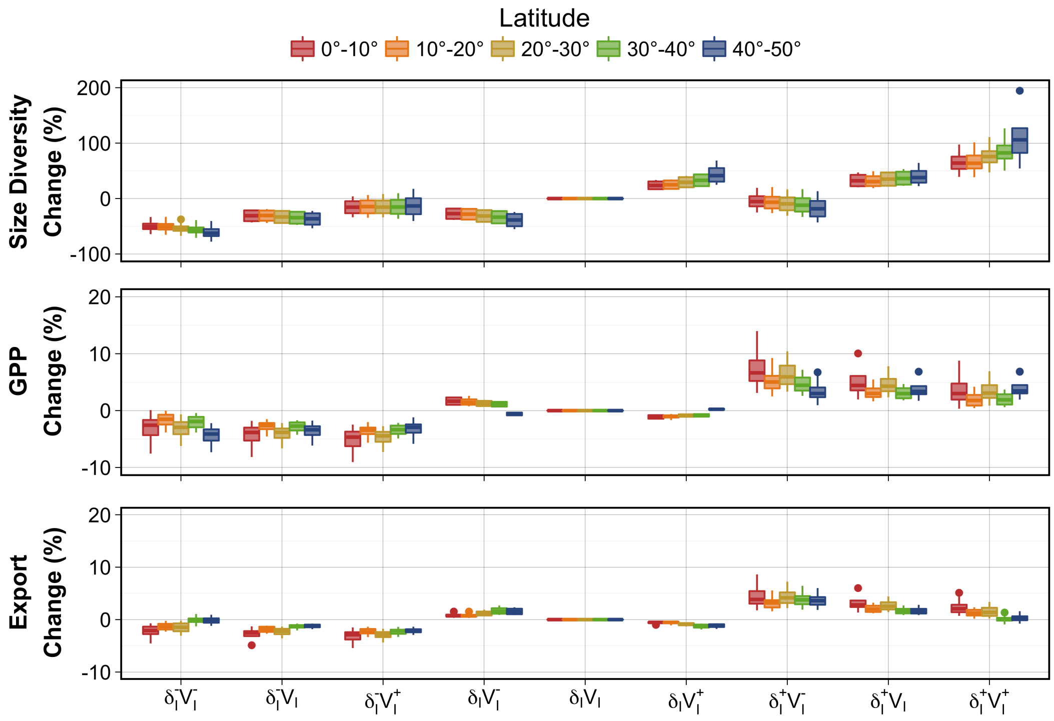

Supplementary Figure 5. Sensitivity test to changes in the parameters δ_I and V_I. has on phytoplankton size diversity, gross primary production (GPP) and export at each location along the Atlantic Meridional Transect. For simplicity, we reduced the 25 possible combinations of changes in ±25% and ±50% into 9 combinations, considering only increase (superscripted plus sign), decrease (superscripted minus sign) or no change (no superscripted sign) of the parameters with respect to their reference values. For guidance see Supplementary Table 1. Notice that latitude is represented in absolute values, therefore, each box-plot includes the results for two ten degree location one in the northern and another in the southern hemisphere, e.g. 40 to 50 °N and -40 to -50 °S.

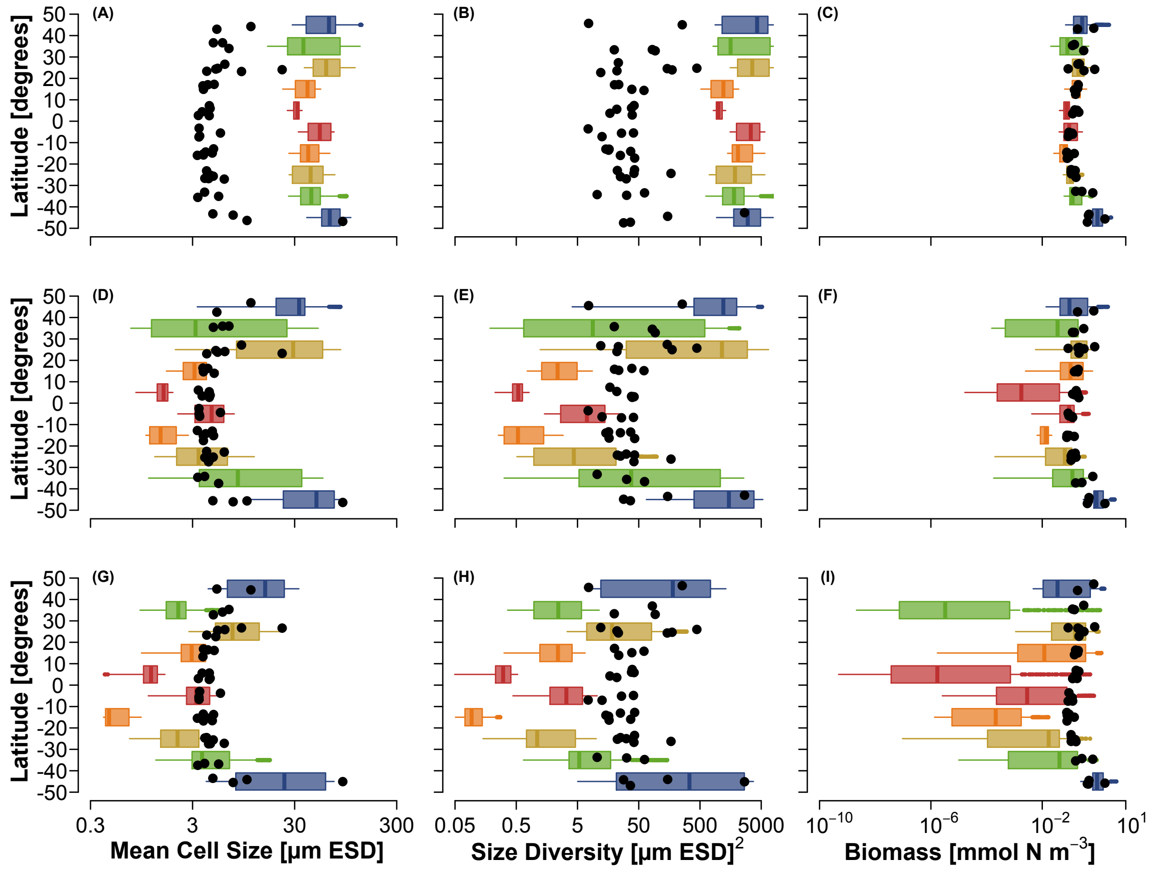

Supplementary Figure 6. Sensitivity of model prediction to fixed mean cell size changes in nutrient uptake. Changes in mean cell size where fixed to 3 (A-B), 10 (D-F), and 30 (G-I) µm ESD. Boxplots represent the temporal variability of a typical annual cycle predicted by the trait-based model at each 10° by 10° location, with vertical lines representing the median, hinges of the box representing the first, and third quartile, whiskers representing the 95% confidence interval, and small coloured dots representing outliers. The black dots represent *in situ* observations.

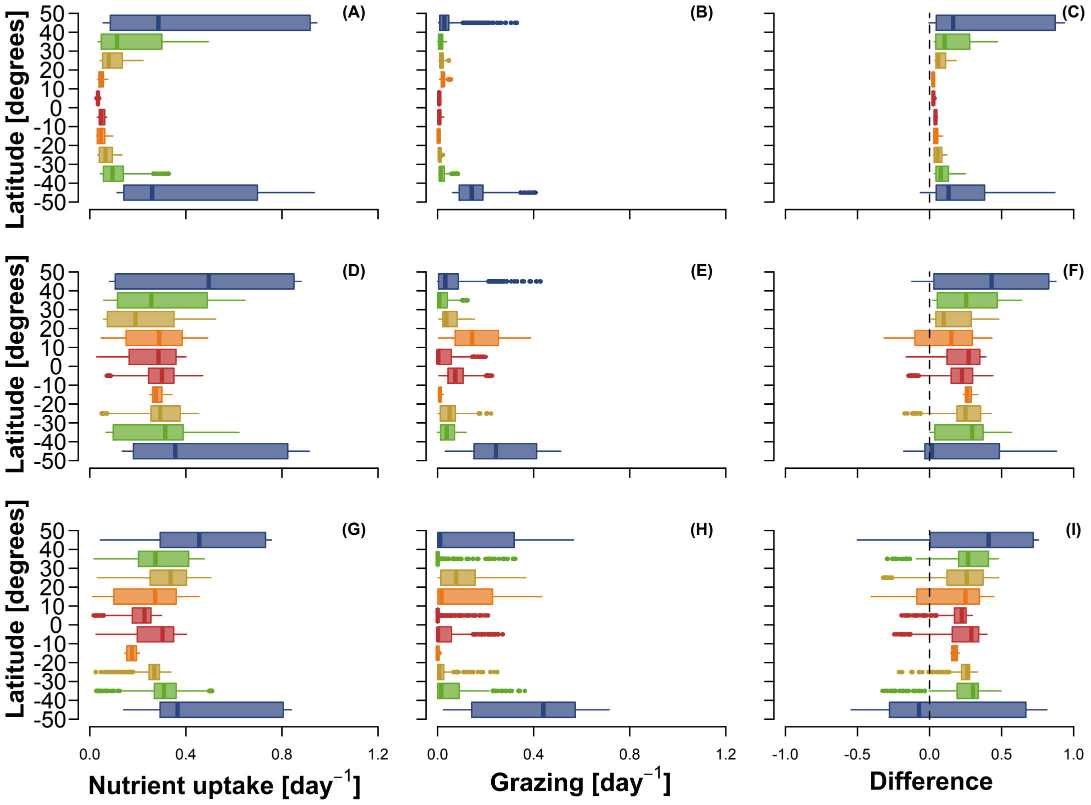

Supplementary Figure 7. Sensitivity of main size dependent processes (nutrient uptake, grazing and their difference) to fixed mean cell size changes in nutrient uptake. Changes in mean cell size where fixed to 3 (A-C), 10 (D-F), and 30 (G-I) μm ESD. Boxplots represent the temporal variability of a typical annual cycle predicted by the trait-based model at each 10° by 10° location, with vertical lines representing the median, hinges of the box representing the first, and third quartile, whiskers representing the 95% confidence interval, and small coloured dots representing outliers. The black dashed line mark the null difference between nutrient uptake and grazing, and where positive values highlights when uptake is greater than grazing and negative values when grazing is greater than uptake.

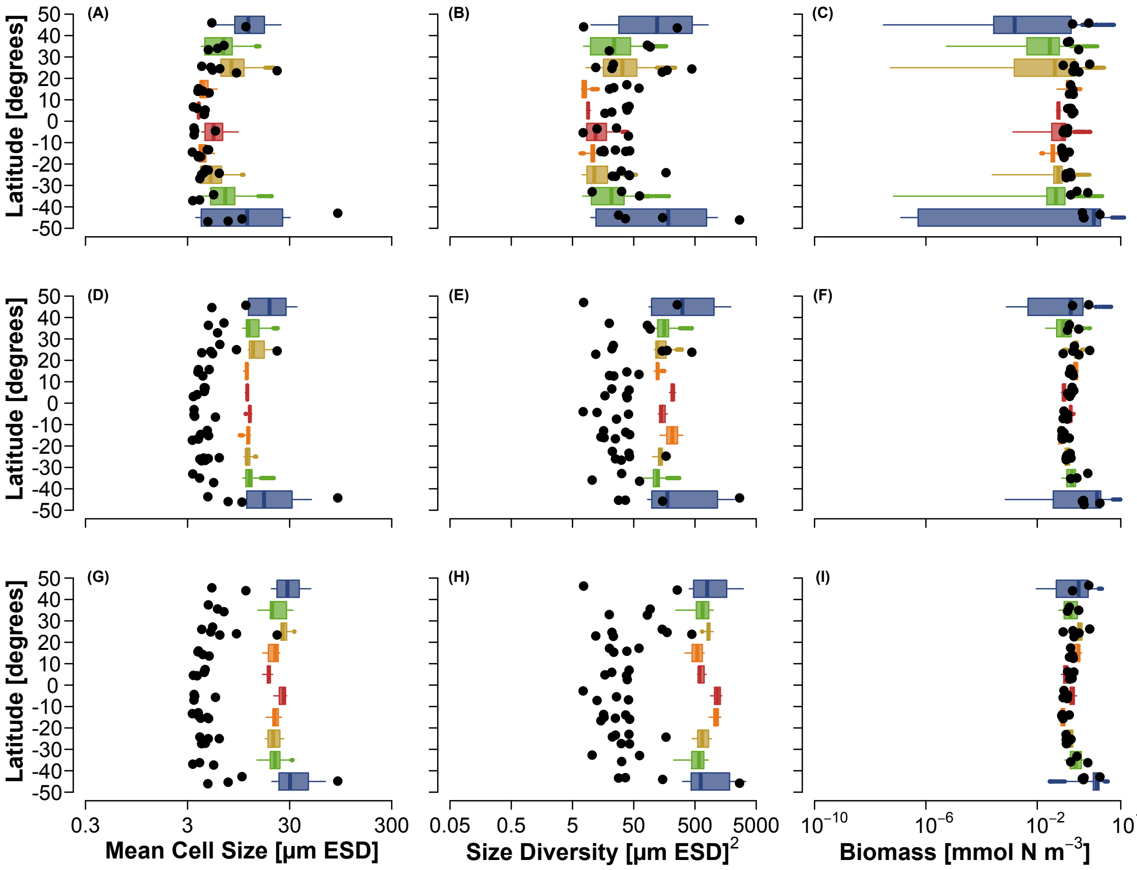

Supplementary Figure 8. Sensitivity of model prediction to fixed mean cell size changes in grazing. Changes in mean cell size where fixed to 3 (A-B), 10 (D-F), and 30 (G-I) µm ESD. Boxplots represent the temporal variability of a typical annual cycle predicted by the trait-based model at each 10° by 10° location, with vertical lines representing the median, hinges of the box representing the first, and third quartile, whiskers representing the 95% confidence interval, and small coloured dots representing outliers. The black dots represent *in situ* observations.

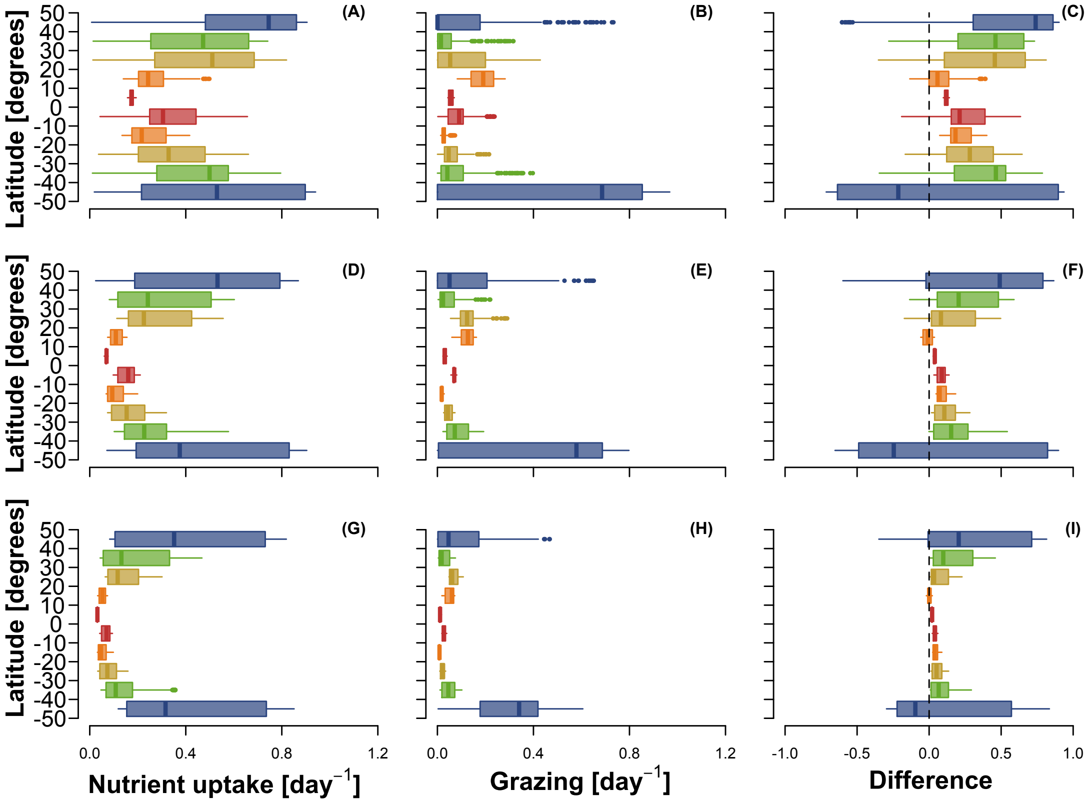

Supplementary Figure 9. Sensitivity of main size dependent processes (nutrient uptake, grazing and their difference) to fixed mean cell size changes in grazing. Changes in mean cell size where fixed to 3 (A-C), 10 (D-F), and 30 (G-I) µm ESD. Boxplots represent the temporal variability of a typical annual cycle predicted by the trait-based model at each 10° by 10° location, with vertical lines representing the median, hinges of the box representing the first, and third quartile, whiskers representing the 95% confidence interval, and small coloured dots representing outliers. The black dashed line mark the null difference between nutrient uptake and grazing, and where positive values highlights when uptake is greater than grazing and negative values when grazing is greater than uptake.

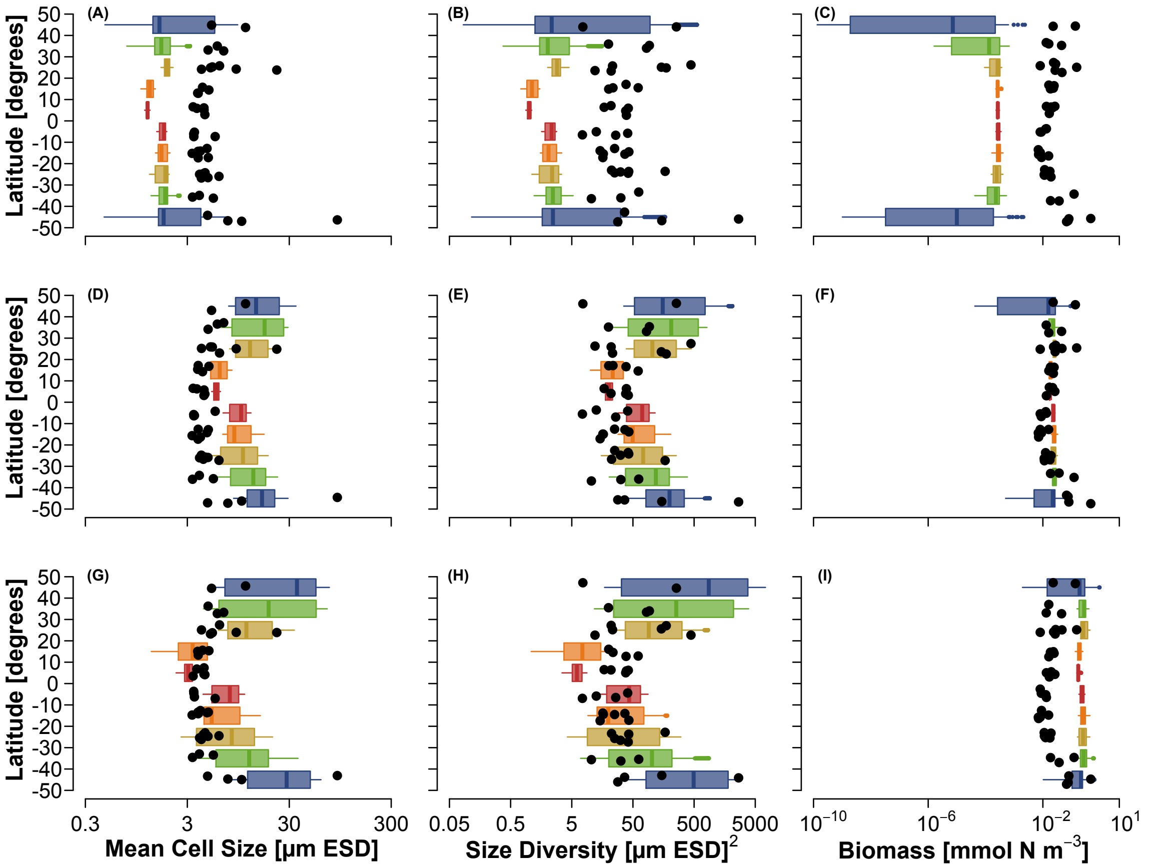

Supplementary Figure 10. Sensitivity of model prediction to fixed nutrient supply of 0.1 (A-B), 2 (D-F), and 10 (G-I) mmol N m^{-3}. Boxplots represent the temporal variability of a typical annual cycle predicted by the trait-based model at each 10° by 10° location, with vertical lines representing the median, hinges of the box representing the first, and third quartile, whiskers representing the 95% confidence interval, and small coloured dots representing outliers. The black dots represent *in situ* observations.

References

1. Acevedo-Trejos E, Brandt G, Smith SL, Merico A. 2016 PhytoSFDM version 1.0.0: Phytoplankton Size and Functional Diversity Model. *Geosci. Model Dev.* **9**, 4071–4085. (doi:10.5194/gmd-2016-102)
2. Fasham MJR, Ducklow HW, Mckelvie SM. 1990 A nitrogen-based model of plankton dynamics in the oceanic mixed layer. *J. Mar. Res.* **48**, 591–639.
3. Evans G, Parslow J. 1985 A model of annual plankton cycles. *Biol. Oceanogr.* **3**, 327–347.
4. Eppley R. 1972 Temperature and phytoplankton growth in the sea. *Fish. Bull.* **70**, 1063–1085.
5. Steele J. 1962 Environmental control of photosynthesis in the sea. *Limnol. Oceanogr.* **7**, 137–150.
6. Ebenhöh W, Barreta-Bekker JG, Baretta J. 1997 The primary production module in the marine ecosystem model ERSEM II, with emphasis on the light forcing. *J. Sea Res.* **38**, 173–193.
7. Monod J. 1949 The growth of bacterial cultures. *Annu. Rev. Microbiol.* **3**, 371–94.
8. Litchman E, Klausmeier CA, Schofield O, Falkowski PG. 2007 The role of functional traits and trade-offs in structuring phytoplankton communities: scaling from cellular to ecosystem level. *Ecol. Lett.* **10**, 1170–1181. (doi:10.1111/j.1461-0248.2007.01117.x)
9. Hansen B, Bjørnsen PK, Hansen PJ. 1994 The size ratio between planktonic predators and their prey. *Limnol. Oceanogr.* **39**, 395–403. (doi:10.4319/lo.1994.39.2.0395)
10. Hansen PJ, Bjornsen PK, Hansen BW. 1997 Zooplankton grazing and growth: Scaling within the 2-2,000-mu m body size range. *Limnol. Oceanogr.* **42**, 687–704. (doi:10.4319/lo.1997.42.4.0687)
11. Gentleman W, Leising A, Frost B, Strom S, Murray J. 2003 Functional responses for zooplankton feeding on multiple resources: a review of assumptions and biological dynamics. *Deep Sea Res. Part II Top. Stud. Oceanogr.* **50**, 2847–2875. (doi:10.1016/j.dsr2.2003.07.001)
12. Acevedo-Trejos E, Brandt G, Bruggeman J, Merico A. 2015 Mechanisms shaping phytoplankton community structure and diversity in the ocean.

Sci. Rep. **5**, 8918. (doi:10.1038/srep08918)

13. Kiørboe T. 1993 Turbulence, phytoplankton cell size, and the structure of pelagic food webs. *Adv. Mar. Biol.* **29**, 1–72.
14. Wirtz KW, Eckhardt B. 1996 Effective variables in ecosystem models with an application to phytoplankton succession. *Ecol. Modell.* **92**, 33–53. (doi:10.1016/0304-3800(95)00196-4)
15. Norberg J, Swaney DP, Dushoff J, Lin J, Casagrandi R, Levin SA. 2001 Phenotypic diversity and ecosystem functioning in changing environments: a theoretical framework. *Proc. Natl. Acad. Sci.* **98**, 11376–81. (doi:10.1073/pnas.171315998)
16. Merico A, Bruggeman J, Wirtz K. 2009 A trait-based approach for downscaling complexity in plankton ecosystem models. *Ecol. Modell.* **220**, 3001–3010. (doi:10.1016/j.ecolmodel.2009.05.005)
17. Fasham MJR. 1993 Modelling the marine biota. In *The Global Carbon Cycle* (ed M Heimann), pp. 457–504. Heidelberg: Springer-Verlag.
18. Fasham MJR, Flynn KJ, Pondaven P, Anderson TR, Boyd PW. 2006 Development of a robust marine ecosystem model to predict the role of iron in biogeochemical cycles: A comparison of results for iron-replete and iron-limited areas, and the SOIREE iron-enrichment experiment. *Deep. Res. Part I Oceanogr. Res. Pap.* **53**, 333–366. (doi:10.1016/j.dsr.2005.09.011)
19. Edwards AM, Brindley J. 1996 Oscillatory behaviour in a three-component plankton population model. *Dyn. Stab. Syst.* **11**, 347–370.
20. Fasham MJR. 1995 Variations in the seasonal cycle of biological production in subarctic oceans: A model sensitivity analysis. *Deep Sea Res. Part I Oceanogr. Res. Pap.* **42**, 1111–1149. (doi:10.1016/0967-0637(95)00054-A)
21. Mitra A, Flynn KJ, Fasham MJR. 2007 Accounting for grazing dynamics in nitrogen-phytoplankton-zooplankton (NPZ) models. *Limnol. Oceanogr.* **52**, 649–661. (doi:10.4319/lo.2007.52.2.0649)
22. Fasham MJR, Boyd PW, Savidge G. 1999 Modeling the relative contributions of autotrophs and heterotrophs to carbon flow at a Lagrangian JGOFS station in the Northeast Atlantic: The importance of DOC. *Limnol. Oceanogr.* **44**, 80–94. (doi:10.4319/lo.1999.44.1.0080)

23. Terseleer N, Bruggeman J, Lancelot C, Gypens N. 2014 Trait-based representation of diatom functional diversity in a plankton functional type model of the eutrophied Southern North Sea. *Limnol. Oceanogr.* **59**, 1–16. (doi:10.4319/lo.2014.59.6.0000)
24. Fuchs H l, Franks PJ. 2010 Plankton community properties determined by nutrients and size-selective feeding. *Mar. Ecol. Prog. Ser.* **413**, 1–15. (doi:10.3354/meps08716)
